# Bendamustine-induced nephrogenic diabetes insipidus – A case report

**DOI:** 10.1177/10781552211013878

**Published:** 2021-05-14

**Authors:** Audrey Desjardins, Viviane Le-Nguyen, Léa Turgeon-Mallette, Chloé Vo, Jean-Samuel Boudreault, Jean-Philippe Rioux, Xue Feng, Amélie Veilleux

**Affiliations:** 1 Faculty of Pharmacy, Université de Montréal, Montreal, Canada; 2 Division of Hematology and Oncology, Department of Medicine, Hôpital du Sacré-Cœur de Montréal, Montreal, Canada; 3 Division of Nephrology, Department of Medicine, Hôpital du Sacré-Cœur de Montréal, Montreal, Canada; 4 Department of Pharmacy, Hôpital du Sacré-Cœur de Montréal, Montreal, Canada

**Keywords:** Bendamustine, diabetes insipidus, nephrogenic diabetes insipidus, adverse effect

## Abstract

**Introduction:**

In patients with relapsed or refractory lymphoma, high-dose chemoimmunotherapy with subsequent autologous hematopoietic cell transplantation (HCT) is a standard of care. Bendamustine, an alkylating agent, is used in the BeEAM (bendamustine, etoposide, cytarabine, melphalan) protocol for conditioning therapy before autologous HCT in patients with relapsed or refractory lymphoma who are eligible for transplant. There is no consensus regarding an optimal salvage regimen and the approach varies according to toxicity.

**Case report:**

We present a case of partial nephrogenic diabetes insipidus after receiving bendamustine, as part of the BeEAM protocol.

**Management and outcome:** The patient was managed with parenteral fluid administration and intranasal desmopressin before the condition resolved on its own.

**Discussion:**

We summarize published reports of bendamustine-induced diabetes insipidus.

## Introduction

Bendamustine is an alkylating agent often used as curative and palliative treatment of B cell Non-Hodgkin and Hodgkin lymphomas, for which it has shown to be very effective.^1–3^ It is also part of the BeEAM protocol (bendamustine, etoposide, cytarabine and melphalan), used in autologous hematopoietic cell transplantation (HCT) for patients with relapsed/refractory lymphoma.^4^ Autologous HCT is associated with a variety of possible complications, including infections secondary to chemotherapy-induced aplasia, and adverse effects of the medication used.^5^ Regarding bendamustine, examples of common adverse effects (≥15%) are nausea, vomiting, fatigue, headache, rash, stomatitis and pancytopenia, and less common ones that can be seen are chest pain, tachycardia and nasal congestion.^6^ A very rare adverse effect that is seldom reported in the literature is nephrogenic diabetes insipidus (NDI).

NDI is defined as the kidneys’ inability to concentrate urine due to antidiuretic hormone resistance (ADH).^7^ It results in severe polyuria causing thirst and polydipsia. Few cases of drug-induced NDI have been published with bendamustine.^8^,^9^ To date, no study in the literature states a clear association between NDI and bendamustine. This adverse event needs prompt recognition for optimal management. In this article, we present a case of a 48-year-old patient who received bendamustine in the BeEAM protocol and subsequently developed partial nephrogenic diabetes insipidus. Written consent was obtained from the patient.

## Case report

Our case concerns a 48-year-old male with a history of syphilis and heterozygous AS sickle cell trait. Mantle cell lymphoma with multiple lymphomatous polyposis had been diagnosed based on a bone marrow biopsy. The patient received three cycles of R-Maxi CHOP and three cycles of R-High Dose-Ara-C (Nordic protocol^10^) with complete response on the CT scan. His peripheral blood stem cells (PBSC) were mobilized and harvested on his last cycle of R-High Dose Ara-C with granulocyte-colony stimulating factor (G-CSF) and plerixafor. Seven months after his diagnosis and two months after his last chemoimmunotherapy, he underwent the BeEAM protocol for an autologous HCT, which consists of bendamustine 200 mg/m^2^ intravenously given on days –8 and –7, cytarabine 200 mg/m^2^ intravenously twice a day on days –6 to –3, etoposide 100 mg/m^2^ intravenously twice a day on days –6 to –3, and melphalan 140 mg/m^2^ intravenously on day –2.^4^ He was only taking vitamin D supplements at the time.

**Figure 1.**
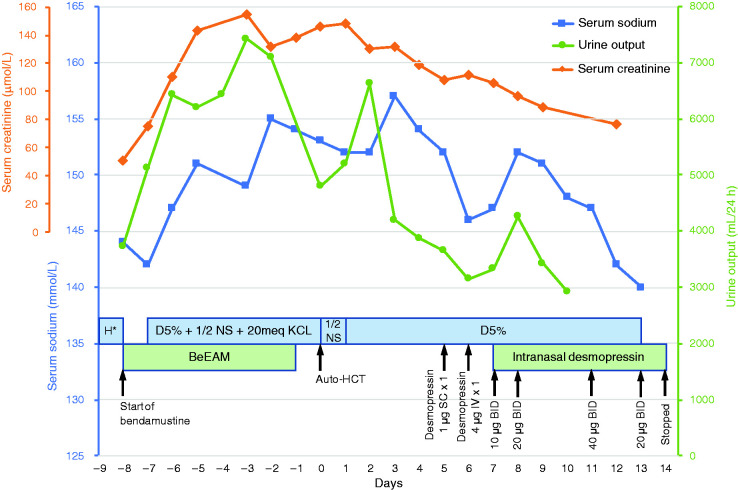
Serum sodium and urine output during hospitalization.H*: D5% + 1/2 NS + KCl 20 mEq alternating with NS + KCl 20 mEq.

Baseline laboratory workup was unremarkable, as shown in Table 1. On day –8, he began the BeEAM protocol and received his first dose of intravenous bendamustine 440 mg (200 mg/m^2^). He also received intravenous fluids as part of the BeEAM protocol. From day –6, his urine output, serum sodium and serum creatinine started to increase (Figure 1). His urine output markedly increased from 3725 mL on day –8 to 7425 mL on day –3 and the serum creatinine increased from 75 µmol/L on day –7 to 155 µmol/L on day –3. His serum sodium also increased to a peak of 155 mmol/L on day –2. During this time, the patient only complained of mild nausea. Vital signs were stable. Forced diuresis with furosemide was suspended and oral hydration was optimized.

**Table 1.** Laboratory results on day –8, day –6 and day 0.

Laboratory test (blood samples)	Day –8	Day –6	Day 0	Normal values
Serum creatinine, µmol/L	71	110	146	55–88
Sodium, mmol/L	144	147	153	136–146
Potassium, mmol/L	4	3.8	5.8	3.5–5.3
Chloride, mmol/L	104	109	118	99–109
Calcium, mmol/L	2.22	2.21	2.07	2.15–2.62
Magnesium, mmol/L	0.78	0.86	0.91	0.70–1.05
Phosphorus, mmol/L	0.96	1.25	1.34	0.80–1.45
Bicarbonate, mmol/L	26.5	24.2	22.2	24.0–31.0
Albumin, g/L	41	42	39	35–50
Glucose, mmol/L	5	6.6	6.9	3.9–6.1

On day –1, the patient started complaining of polyuria, nocturia and thirst. However, he could not drink, since he had severe mucositis and odynophagia due to his chemotherapy, which led him to be put on total parenteral nutrition a few days after. A nephrology consultation request was made. Laboratory studies revealed the following values: random urine sodium 56 mmol/L; random urine osmolality 307 mOsm/kg and specific urine gravity from urinalysis 1.006. Despite the normal range of urine osmolality, the nephrologist team suspected a nephrogenic diabetes insipidus (NDI) along with concomitant dehydration in absence of other identified causes of polyuria. However, no water deprivation test or desmopressin challenge was done since the patient had developed an acute kidney injury (AKI), and required fluids for his hypernatremia and his upcoming HCT. After the HCT, the patient’s infusion was first switched to 0.45% sodium chloride and subsequently to dextrose 5%. Serum sodium levels showed little improvement while fluid balance improved but remained negative. On day 5, desmopressin 1 mcg was administered subcutaneously with no effect on diuresis. The following day, a desmopressin (4 mcg) challenge was administered intravenously and showed a 33% increase from baseline urine osmolality of 204 mOsm/kg to an average of 277 mOsm/kg with a decrease in diuresis. These values were compatible with a diagnosis of diabetes insipidus. The patient remained polyuric and highly dependent on fluids due to his hypernatremia. Since he was partially responsive to the desmopressin challenge, intranasal desmopressin was started on day 8. The dose was increased until it reached desmopressin 40 mcg twice daily intranasally on day 11 (Figure 1). A brain magnetic resonance imaging (MRI) was then performed on day 12 to rule out central lymphomatous infiltrates, which came back negative and therefore reinforced a diagnosis of partial NDI. On day 13, his natremia returned to normal range along with diuresis decreased to 600-800 cc/8 h, intravenous dextrose 5% was stopped. Desmopressin was weaned off and then stopped on day 15. By then, the patient’s nocturia had markedly improved and NDI was considered resolved. On day 18, the patient was discharged from the hospital. To this day, he remains in remission of his lymphoma.

## Discussion

### Diabetes insipidus

Diabetes insipidus (DI) is a condition where water homeostasis by vasopressin is impaired. It can be caused by a decreased vasopressin secretion (central diabetes insipidus) or the kidney’s resistance to the vasopressin action (nephrogenic diabetes insipidus).^11^ In either case, the kidneys are unable to fully concentrate the urine and urine osmolality is low. Thus, patients with DI show symptoms of polyuria, nocturia and polydipsia.^11^ Serum osmolality and natremia can be within normal limits if the patient can drink enough to compensate for the water loss.^12,13^ A urine osmolality below 300 mOsm/kg along with serum hyperosmolality and polyuria suggests the presence of DI.^11,12^ To diagnose DI or to differentiate between types of DI, a water deprivation test with the administration of desmopressin can be done. The patient is placed under water restriction to stimulate the secretion of endogenous vasopressin, and its impact on urine concentration is measured through urine osmolality. In DI, urine osmolality remains low since endogenous vasopressin action is deficient.^12^ Desmopressin is then administered, with urine osmolality measured before and after. Since central DI is caused by a lack of vasopressin, the administration of desmopressin will have a significant impact on urine osmolality, with elevations from 100 to 800% in complete central diabetes insipidus (CDI) and 15 to 50% in partial CDI.^14^ As for nephrogenic diabetes insipidus (NDI), kidneys are resistant to vasopressin and desmopressin will have little to no effect on urine osmolality. However, many patients are only partially resistant to ADH and may respond with an up to 45% elevation in urine osmolality.^14^

Both CDI and NDI can be acquired or inherited. Common acquired causes of CDI include neoplasms, trauma and neurosurgery. Most of these causes can be identified from the patient’s history or the brain MRI.^12^ Common acquired causes of NDI include drugs, metabolic disturbances, such as hypercalcemia and hypokalemia, and renal disease.^12^ In these cases, the condition is often reversible with cessation of the causative drug or correction of the metabolic disturbance.^15^ Both CDI and NDI may also be idiopathic. The most common drug to cause NDI is lithium. Other involved pharmacological classes are antibiotics, antiviral agents, antifungal agents, antineoplastic agents (cyclophosphamide, ifosfamide) and antipsychotics.^15–17^ Treatments include sodium restriction and desmopressin for CDI. Thiazides and NSAIDs can also be considered for chronic types of NDI. In any case, appropriate fluid intake is essential and correction of the underlying cause should be sought whenever possible.^11–13^

### Case management

DI was suspected with the presentation of polyuria, thirst and low urine osmolality. A partial NDI diagnosis was made based on the desmopressin challenge resulting in an increase of 33% in urinary osmolality from baseline. A water deprivation test was not performed to confirm the diagnosis since the patient had just received chemotherapy and had developed an AKI. DI can be a manifestation of primary or secondary brain tumors involving the hypothalamic-pituitary region. However, pituitary involvement is rare in lymphomas and is usually seen on the brain MRI.^18^ Moreover, HCT would not have been performed in patients with central nervous system infiltrates in mantle cell lymphoma. In our case, the brain MRI was negative for any lymphoma infiltrate and CDI was ruled out.

Kidney injuries, including acute tubular necrosis and ureteral obstruction, are potential causes of acquired NDI.^12^ In our case, the patient developed an AKI in the days following bendamustine administration. Dose-intensified bendamustine in HCT conditioning regimens has been associated with all-grade AKI.^19–21^ Besides bendamustine, the patient was not exposed to any other drugs known for causing AKI or nephrogenic DI at the time of presentation. Although he had received three doses of cyclophosphamide as part of the Nordic protocol, his last dose of cyclophosphamide was administered two months earlier. Furthermore, the timing between the administration of bendamustine and the onset of dilute polyuria consisted of a few days, similar to other cases in the literature.^8,9^ This suggests bendamustine was the cause of NDI and not cyclophosphamide. The drug was already discontinued at the time of DI diagnosis and there was no rechallenge. NDI also resolved without needing long-term treatment, which points towards the cause being an adverse drug reaction. The Naranjo adverse drug reaction score reveals a score of 7 which indicates a probable adverse drug reaction (Supplemental Material 1. Naranjo Algorithm Assessment).^22^ Of note, individuals with sickle cell trait are at risk for various renal complications such as hyposthenuria, a defect in the capacity to concentrate urine.^23,24^ If present, this condition could have contributed to our patient’s presentation.

As mentioned, a crucial part of treating nephrogenic DI is to correct the underlying cause. In our case, bendamustine was suspected early in the process. However, the only two bendamustine doses in the BeEAM protocol had been administered by the time the diagnosis was made, so the underlying cause was already corrected. As for the management of hypernatremia and AKI, oral hydration was difficult since the patient had severe odynophagia due to his chemotherapy. Intravenous 0.45% saline was therefore administered and later switched to dextrose 5%, which are both appropriate in the context of hypernatremia. The rate of fluid administration was revised daily. Furthermore, desmopressin was used to reduce polyuria associated with DI and to limit high fluid intake. Desmopressin is usually not the first line of treatment for nephrogenic DI as patients tend to be resistant to its effects, thus requiring higher doses.^12^ However, our patient demonstrated a partial response when the desmopressin challenge was administered (the urine osmolality increased by 33%). Therefore, we considered it was beneficial to continue with intranasal desmopressin in this case, especially as thiazides and NSAIDS were not appropriate due to AKI.

### Mechanism

Bendamustine is an alkylating agent that has three structural elements: a 2-chloroethylamine alkylating group, a benzimidazole ring, and a butyric acid side chain.^25^ It acts by alkylating and crosslinking DNA strands, causing cell death via several pathways.^6^ There are case reports of Fanconi syndrome combined with NDI that are caused by ifosfamide, another alkylating agent which also acts via a chloroethylamine group.^26–30^ The mechanism by which ifosfamide causes NDI is unclear, but the hypokalemia associated with impaired proximal tubular function might be a contributing factor. It is thought that acquired forms of NDI are due to defects or reduced expression of aquaporin 2, a vasopressin-regulated water channel of the collecting duct, but they appear to be reversible after discontinuation of the causing agent.^31^ Bendamustine could have caused NDI in our patient by a similar mechanism. However, bendamustine does not seem to cause Fanconi syndrome.

### Literature review

We have only found two published case reports on NDI involving bendamustine (Table 2). The first case is a 59-year-old male who developed partial NDI following the first cycle of bendamustine and dexamethasone for light chain amyloidosis.^8^ The second case is a 59-year-old male who developed NDI within days of completing his sixth cycle of rituximab and bendamustine for chronic lymphocytic leukemia.^9^ Our patient’s presentation is similar to these case reports found in the literature. All three cases had an onset of DI symptoms within a few days of taking bendamustine, although the case by Derman et al (2017) only developed DI after the sixth cycle. Our case is most similar to the case report by Uwumugambi et al (2016), in which the patient was diagnosed with partial NDI and where toxicity was reversible. The patient was initially treated with a low sodium diet and hydrochlorothiazide 12.5 mg daily orally, with only a modest improvement in polyuria. Intranasal desmopressin was then initiated which resulted in a drastic improvement in symptoms. The patient was kept on bendamustine and after four more cycles without any recurrence, desmopressin and HCTZ were discontinued. From these case reports, it suggests that longer exposure to the offending drug lengthens the time required for recovery. This trend was also noted in a systematic review on causes of reversible NDI.^15^

**Table 2.** Summary of the case reports on bendamustine-induced NDI.

	Uwumugambi et al. (2016)	Derman et al. (2017)	Our case report
Disease	Amyloid light-chain amyloidosis	Chronic lymphocytic leukemia	Mantle cell lymphoma
Onset of DI symptoms	Two days after starting first cycle	Within days of completing sixth cycle	Polyuria three days after first dose
Na (mEq/L)	148	163	152
uOsm (mOsm/kg)	207	145	204
uNa (mEq/L)	69	34	41
Specific urine gravity	1.003	1.005	1.006
Hydration		D5W	½ NS-D5 then D5W
Urine output (L) / day	9	10.175	3.65
Water deprivation test	uOsm unchanged	Not done	Not done
Desmopressin challenge	36% increase from baseline	uOsm unchanged	33% increase from baseline
Management	Low sodium diet HCTZ 12.5 mg po daily Intranasal desmopressin (0.01%) daily	Sodium restriction HCTZ 25 mg po daily	Intranasal desmopressin (0.01%) twice daily
Resolution	Received four more cycles of bendamustine, treatments ceased with no recurrence	Remained symptomatic after two months	Resolved after 8-day course of desmopressin (17 days after first suspicion)

D5W: dextrose 5%; NS: normal saline; die: once daily; bid: twice daily; HCTZ: hydrochlorothiazide; uOsm: urine osmolality.

## Conclusion

Although rare cases of bendamustine-related diabetes insidipus have been described, this report is the first involving BeEAM conditioning preceding autologous HSCT.^8^,^9^ Despite the rarity of this entity, prompt treatment is essential. In our patient’s case, it occurred a few days after he received bendamustine, and presented itself as partial nephrogenic diabetes insipidus with polyuria and urinary osmolality below 300 mOsm/kg. Our patient was then treated with intranasal desmopressin along with intravenous fluids. His NDI was resolved after eight days of treatment. The patient had a successful HCT, and he remains in remission of his disease. Further studies are needed to clarify the mechanism by which bendamustine causes NDI and to identify potential risk factors. NDI should be suspected in patients exposed to bendamustine who subsequently develop hypernatremia and polyuria. We recommend treating NDI according to the best available evidence.
